# Minimally Invasive Rectal Surgery: Current Status and Future Perspectives in the Era of Digital Surgery

**DOI:** 10.3390/jcm14041234

**Published:** 2025-02-13

**Authors:** Marta Goglia, Matteo Pavone, Vito D’Andrea, Veronica De Simone, Gaetano Gallo

**Affiliations:** 1 Department of Medical and Surgical Sciences and Translational Medicine, School in Translational Medicine and Oncology, Faculty of Medicine and Psychology, Sapienza University of Rome, 00185 Rome, Italy; martagoglia@hotmail.com; 2 UOC Ginecologia Oncologica, Dipartimento di Scienze per la Salute della Donna e del Bambino e di Sanità Pubblica, Fondazione Policlinico Universitario A. Gemelli, Istituto di Ricovero e Cura a Carattere Scientifico (IRCCS), 00168 Rome, Italy; matteopavone.21@gmail.com; 3 IHU Strasbourg, Institute of Image-Guided Surgery, 67000 Strasbourg, France; 4 IRCAD, Research Institute against Digestive Cancer, 67000 Strasbourg, France; 5 Department of Surgery, Sapienza University of Rome, 00185 Rome, Italy; vito.dandrea@uniroma1.it; 6 Proctology and Pelvic Floor Surgery Unit, Ospedale Isola Tiberina-Gemelli Isola, 00186 Rome, Italy; veronicadesimone@libero.it

**Keywords:** minimally invasive surgical procedures, inflammatory bowel diseases, rectal cancer, TEM, TAMIS, robotic surgery, total mesorectal excision

## Abstract

Over the past two decades, minimally invasive approaches in rectal surgery have changed the landscape of surgical interventions, impacting both malignant and benign pathologies. The dynamic nature of rectal cancer treatment owes much to innovations in surgical techniques, reflected in the expanding literature on available treatment modalities. Local excision, facilitated by minimally invasive surgery, offers curative potential for patients with early T1 rectal cancers and favorable pathologic features. For more complex cases, laparoscopic and robotic surgery have demonstrated significant efficacy and provided precise, durable outcomes while reducing perioperative morbidity and enhancing postoperative recovery. Additionally, advancements in imaging, surgical instrumentation, and enhanced recovery protocols have further optimized patient care. The integration of multidisciplinary care has also emerged as a cornerstone of treatment, emphasizing collaboration among surgeons, oncologists, and radiologists to deliver personalized, evidence-based care. This narrative review aims to elucidate current minimally invasive surgical techniques and approaches for rectal pathologies, spanning benign and malignant conditions, while also exploring future directions in the field, including the potential role of artificial intelligence and next-generation robotic platforms.

## 1. Introduction

Colorectal cancer (CRC) ranks as the third most prevalent malignancy worldwide, contributing significantly to cancer-related mortality [1,2]. Rectal cancer alone accounts for about 30% of CRC cases, needing a multidisciplinary management approach focused on tumor location, staging, and surgical resectability [3,4]. Innovations in surgical techniques have dynamically transformed treatment paradigms, notably through the introduction of approaches to minimally invasive surgery (MIS).

The most frequent histological type of CRC is adenocarcinoma, originating from adenomatous polyps in a well-documented adenoma-carcinoma sequence. Approximately 10% of these adenomas evolve into adenocarcinomas over a decade, influenced by genetic pathways such as the adenomatous polyposis coli (APC) pathway, hereditary nonpolyposis colorectal cancer (HNPCC) pathway, and pathways associated with inflammatory bowel disease (IBD), like ulcerative colitis [5,6].

The APC pathway, for instance, starts with the inactivation of the APC gene, leading to unchecked cellular replication and subsequent genetic mutations, including K-RAS and p53, which further drive the cancer progression. In contrast, the HNPCC pathway involves defects in DNA mismatch repair genes (hMLH1, hMSH2, hPMS1, hPMS2, and hMSH6), leading to replication errors found in the majority of HNPCC and a significant portion of sporadic cases. Moreover, the tumor microenvironment, meaning various types of cells, like cancer-associated fibroblasts (CAFs), tumor-associated macrophages, regulatory T cells, and myeloid-derived suppressor cells, play a functional and structural role in the physiology and pathophysiology of the disease. Indeed, the tumor microenvironment can influence the efficacy of molecular-targeted therapies [7,8,9].

Among these genetic and molecular insights, the clinical approach to rectal cancer has also evolved.

The introduction of total mesorectal excision (TME) in the 1980s significantly reduced locoregional recurrence rates and improved survival outcomes when combined with neoadjuvant chemoradiotherapy [10]. Techniques like Transanal Endoscopic Microsurgery (TEM), introduced by Buess et al. in 1985, have facilitated local resections of early-stage cancers, minimizing risks such as anastomotic leakage [11,12].

Laparoscopic advancements have further modified rectal cancer surgery by minimizing hospital stays and enhancing recovery through less invasive procedures in all surgical realities, from high-referral city centers to rural ones [13]. These include the development of multiport to single-incision laparoscopic surgery and Transanal Minimally Invasive Surgery (TAMIS), underscoring a shift toward procedures that maintain oncological safety while reducing surgical trauma. Moreover, recent evidence from the literature emphasizes the importance of timely surgical intervention for CRC, particularly in patients with locally advanced rectal cancer who exhibit poor or no response to neoadjuvant chemoradiotherapy (CRT). The study found that delaying surgery after CRT is linked to worse overall survival (OS) and disease-free survival (DFS) rates. These findings suggest that early re-evaluation and prompt surgical action are crucial for improving outcomes in these patients, challenging the current practice of using the pathological complete response (pCR) as the primary endpoint in treatment strategies [14].

However, an essential aspect of CRC management is early diagnosis, which serves as a cornerstone for optimizing therapeutic approaches and improving oncological outcomes. Advanced diagnostic methods, such as endoscopy, allow for accurate early staging, enabling the timely application of minimally invasive surgical techniques. Indeed, these approaches have demonstrated the best clinical outcomes, particularly in patients diagnosed early, maximizing cure rates while minimizing postoperative morbidity.

This review aims to elucidate the variety of the current minimally invasive techniques for rectal pathologies, assessing their advantages and limitations in an era where such surgical innovations are pivotal. The ongoing debate over the optimal surgical approach highlights the necessity to balance technique-specific benefits against individual patient needs, underlining the complexity of choosing the most suitable surgical strategy in rectal cancer treatment.

### Rectal Cancer and Inflammatory Bowel Disease (IBD)

Rectal cancer predominantly manifests as adenocarcinomas, constituting about 98% of these cancers, with rarer types, like lymphoma (1.3%), carcinoid (0.4%), and sarcoma (0.3%), also present. Symptoms often include bleeding, yet many cases are asymptomatic and found during routine screenings. The multifaceted nature of rectal cancer treatment necessitates a multidisciplinary approach that incorporates surgery, medical oncology, and radiation therapy to optimize patient outcomes [4,15].

Diagnostic protocols for rectal cancer involve a combination of digital rectal examination (DRE) to assess tumor size and mobility, colonoscopy for tumor localization and biopsy, and advanced imaging techniques, such as rectal MRI and endorectal ultrasonography (ERUS). These imaging methods are crucial for evaluating tumor depth and the integrity of surrounding structures, which help guide decisions on neoadjuvant treatments aimed at minimizing local recurrence [16].

For early-stage rectal cancer (cT1 Sm1 N0), less invasive surgical options, like TEM, can offer curative treatment while preserving anorectal function. Alongside TEM, techniques such as transanal excision (TAE) and TAMIS minimize surgical trauma and postoperative complications, thus maintaining quality of life [12,17].

While this review focuses on MIS for rectal cancer, it is important to highlight the distinct therapeutic considerations between sporadic rectal cancer and ulcerative colitis (UC)-related cases. UC-related rectal cancer often arises in the context of chronic inflammation, necessitating tailored management strategies. Unlike sporadic adenocarcinomas, the therapeutic approach for UC patients frequently integrates proctocolectomy with restorative ileal pouch–anal anastomosis (IPAA) to address both cancer and the underlying disease. These patients typically require comprehensive surgical planning to balance oncological safety with long-term quality of life. While 5-ASA remains foundational in the medical management of UC, many patients with moderate-to-severe disease now rely on biologics and immunomodulators to achieve and maintain remission. Moreover, surgery remains pivotal for those developing dysplasia or cancer, particularly after eight years of disease onset when the cancer risk significantly increases. The inclusion of Crohn’s disease (CD) in the discussion expands the scope of MIS applications. However, it is notable that CD management increasingly emphasizes medical therapies, such as biologics, in a top-down strategy, reducing the frequency of surgical interventions. Surgery is often reserved for complications or refractory cases, with MIS providing significant advantages in terms of reduced postoperative morbidity.

Indeed, the advancement of MIS has significantly impacted the management of both rectal cancer and IBD, providing benefits like shorter recovery times, decreased hospital stays, and improved cosmetic outcomes. These techniques adhere to oncological principles as open surgeries but reduce physical trauma, making them crucial for treating T1 and T2 rectal cancers where balancing effective cancer control and morbidity is essential [18].

The surgical management of IBD has changed with the advent of MIS, such as laparoscopic and robotic-assisted surgeries, which are particularly advantageous for managing complex cases characterized by strictures, fistulas, and recurrent disease [19]. These approaches not only reduce the invasiveness of procedures but also help to preserve gastrointestinal function and enhance patient quality of life (QoL).

Patients with IBD frequently face complications, like anemia, malnutrition, and immunosuppression, increasing the surgical risk. MIS, in this context, not only alleviates the physical burden but also provides high-value care by improving patient outcomes and satisfaction.

The surgical strategies for managing IBD may include procedures such as the formation of an ileal J-pouch with IPAA, commonly employed in refractory UC and familial adenomatous polyposis (FAP). This procedure is often performed in steps to ensure safety and optimal results, reflecting the continual evolution of surgical techniques from open to laparoscopic and robotic-assisted methods, showcasing ongoing advancements in medical technology and surgical expertise [20].

## 2. Laparoscopy

MIS, especially laparoscopy, has transformed surgical practices by minimizing intraoperative injury and providing less invasive alternatives to traditional methods. The origins of laparoscopic surgery date back to 1901 in Dresden with Kelling, evolving significantly to the first laparoscopic appendectomy by Semm in 1981 [21]. The technique gained widespread adoption with the introduction of laparoscopic cholecystectomy in 1987 [22].

Laparoscopy includes hand-assisted laparoscopic surgery (HALS), totally intracorporeal laparoscopic surgery (TILS), single-incision laparoscopic surgery (SILS), and robotically assisted laparoscopic surgery (RALS). These innovations have enhanced surgical precision and quality, reducing postoperative pain, shortening recovery times, and improving cosmetic outcomes [23].

The first laparoscopic colon resection was reported in 1991 [24]. Subsequent trials affirmed the equivalence of laparoscopic and open procedures in surgical and oncological outcomes, such as resection margins and lymph node harvests, with additional benefits, including faster recovery of bowel function and reduced blood loss.

However, laparoscopic approaches to rectal cancer pose challenges, especially in patients with a narrow pelvis or obesity, where the bulky mesorectum complicates procedures. Yet, substantial evidence supports its short-term benefits without compromising the oncologic outcomes [25]. Notable trials like COLOR II and COREAN have shown similar results in key metrics like the completeness of resection and locoregional recurrence rates between laparoscopic and open surgery [26,27].

**Figure 1:**
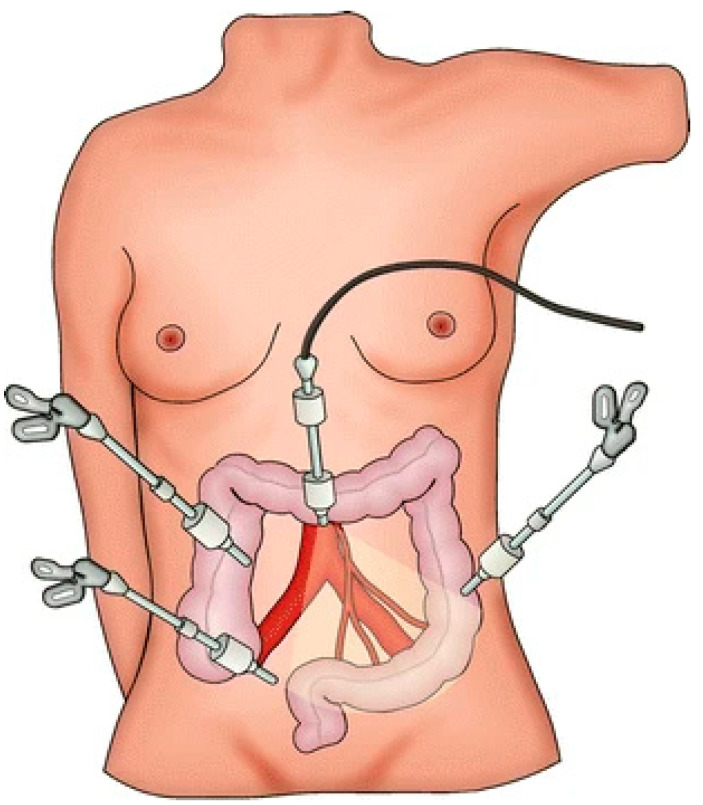
Trocar position of laparoscopic rectal cancer surgery. From Fürst A et al. [28].

The debate on the optimal surgical approach for rectal cancer continues, informed by trials such as ACOSOG Z6051 and AlaCaRT, which explore the non-inferiority of laparoscopic methods. Despite some trials that do not meet the non-inferiority criteria, laparoscopic surgery is widely accepted as safe for rectal cancer (Figure 1) [28], showing no significant differences in disease-free survival or local recurrence compared to open surgery [29,30]. Moreover, laparoscopy plays a crucial role in the management of complex IBD, like CD and UC, particularly for patients resistant to medical treatments like corticosteroids and anti-TNF therapy. Despite technical complexities, laparoscopic approaches for recurrent disease are considered safe, offering outcomes comparable to open surgery with reduced complication rates and shorter hospital stays [31].

In emergencies such as a subtotal colectomy for severe colitis, laparoscopy is advantageous, with outcomes favoring quicker bowel function return, though with longer operative times. Further research supports these postoperative benefits, demonstrating reduced hospital stays and fewer wound infections [32]. This evolution of laparoscopic techniques continues to profoundly impact surgical practices, enhancing patient outcomes across a range of procedures, particularly for CRC and IBD.

### Single-Incision Laparoscopic Surgery (SILS)

**Figure 2:**
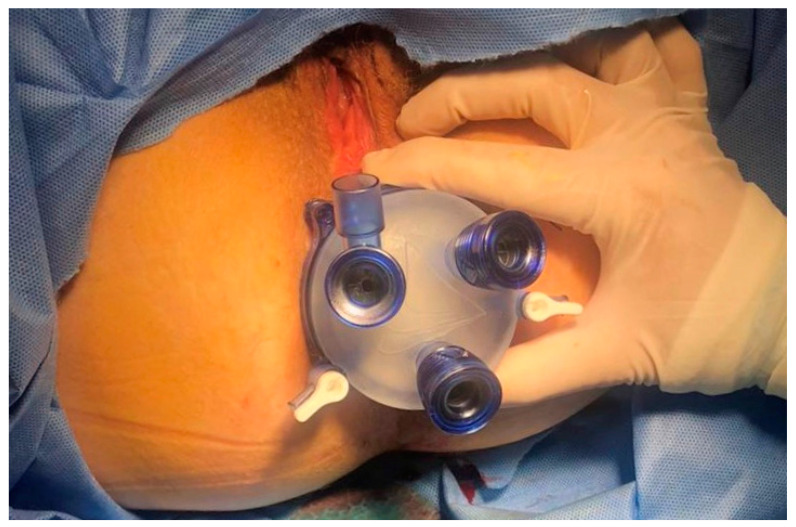
The Gel POINT Path^®^ transanal access platform (Applied Medical) (Gallo G Personal Database).

SILS marks a significant advancement in MIS, promoting enhanced cosmesis and potentially reducing postoperative pain. Introduced in the early 1990s, SILS began with procedures like appendectomy and has then expanded into more complex surgeries, including colorectal resections. The first recorded instance of SILS was a transumbilical appendectomy by Pelosi et al. in 1992, followed by a single-incision transumbilical cholecystectomy by Navarra et al. in 1997 [33,34]. These pioneering efforts set the stage for advancements in colorectal surgery, where SILS was first applied to a radical right hemicolectomy in 2008 by Bucher et al. [35]. The development of SILS has benefited from the introduction of specialized access devices approved by the U.S. Food and Drug Administration, such as the Triport+, which facilitates safe entry into the abdominal cavity and enhances specimen retrieval. Other devices like the QuadPort+ and GelPOINT have further expanded the capabilities of SILS, allowing for major abdominal surgeries through single-incision portals (Figure 2) [36].

A systematic review and meta-analysis by Arezzo et al., encompassing around 1000 patients, highlighted that SILS offers comparable morbidity and parietal complications to conventional laparoscopy. Despite longer operative times, the advantages of improved cosmetic outcomes and reduced postoperative discomfort were significant [37]. These findings prompted further research, including a multicenter randomized trial to better evaluate the safety and efficacy of SILS compared to traditional laparoscopic techniques [38]. This technique has been particularly noted for its application in procedures like anterior rectal resection and total proctocolectomy with ileal pouch–anal anastomosis, where it offers significant cosmetic benefits and reduces the number of incisions, thereby potentially lessening postoperative pain and enhancing early recovery.

Indeed, in terms of colorectal surgery for IBD, SILS has shown promise, especially in elective settings. Studies comparing single-incision ileocolic resections for CD with multi-trocar approaches have found comparable outcomes in terms of postoperative morbidity and pain. For example, a study by Geisler et al. reported initial experiences with SILS proctocolectomy for ulcerative colitis, highlighting its technical feasibility despite the complex nature of the cases [39].

However, the adoption of SILS remains cautious, particularly due to its technical demands and the learning curve associated with its use. Critics argue that the morbidity associated with additional small ports in conventional laparoscopy is negligible, questioning the justification for SILS despite its increased technical demands [40,41].

Furthermore, the potential for reduced postoperative pain and enhanced recovery within enhanced recovery programs suggests that SILS could play a significant role in the future of MIS. While the long-term benefits of SILS still require further investigation, its ability to minimize surgical trauma while maintaining the advantages of traditional laparoscopy positions it as a valuable surgical option. As the technique matures and more data become available, SILS is likely to find broader acceptance and application across a range of surgical disciplines.

## 3. Robotic-Assisted Laparoscopic Surgery (RALS)

**Figure 3:**
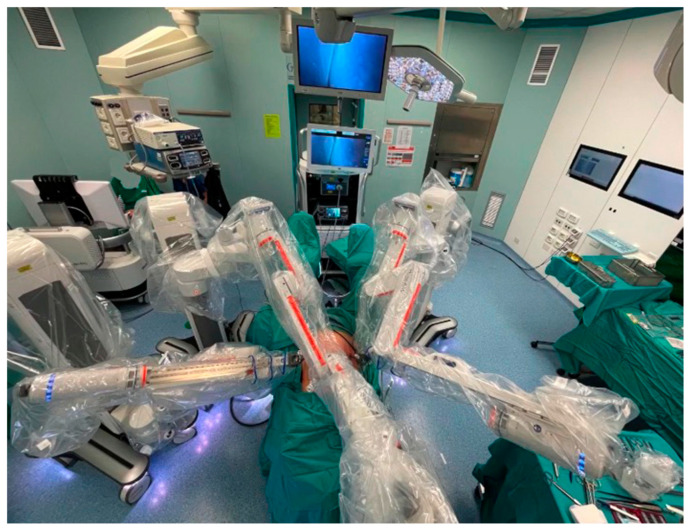
Operating room setting—HUGO RAS system (Medtronic). From Panico et al. [45].

**Figure 4:**
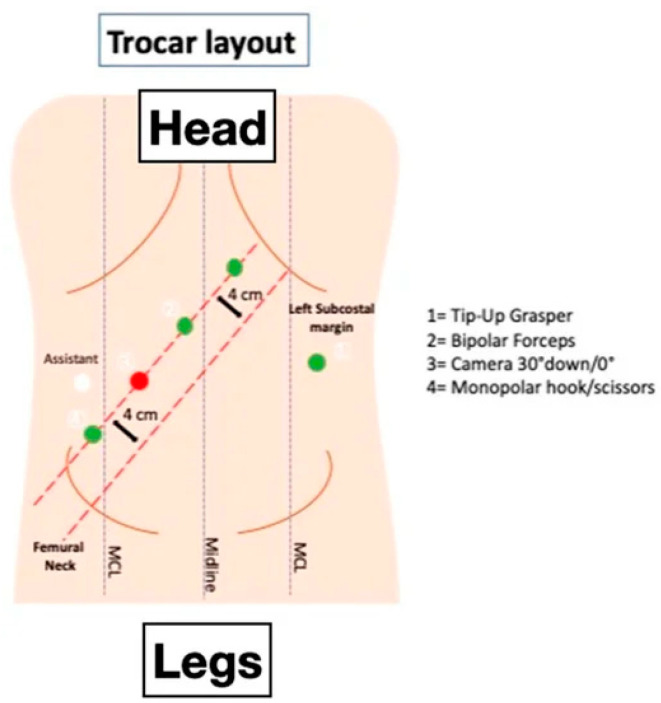
Trocar layout for a fully robotic TME performed with the Davinci Xi Surgical Platform (Intuitive Surgical Inc., Sunnyvale, CA, USA). From Giuratrabocchetta et al. [46].

**Figure 5:**
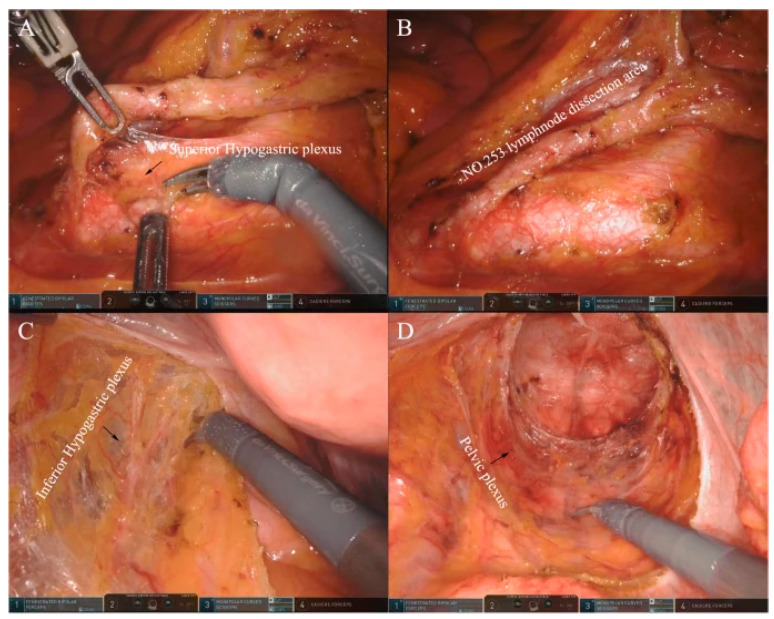
Neuroprotection in robotic TME surgery. (**A**,**B**) Protection of the inferior epigastric nerve during the group 253 lymph node dissection. (**C**) Protection of the inferior epigastric nerve during complete resection of the mesorectum. (**D**) Protection of the pelvic nerve during complete resection of the mesorectum. From Liu G et al. [47].

RALS represents a significant advancement in surgical technology, blending the rapid recovery benefits of laparoscopy with the enhanced visibility and precision of open surgery [38]. After two decades with Da Vinci (Intuitive, California) as the dominant player in the marketplace [42,43,44], several new robotic platforms have emerged, each with distinct characteristics (Figure 3, Figure 4 and Figure 5) [45,46,47].

Robotic systems have been shown to be just as effective as traditional open and laparoscopic techniques for tasks like lymph node harvesting and maintaining negative radial margins in TME [48]. Case-matched analyses of mid- to low-rectal cancer surgeries using robotic, laparoscopic, or open approaches have also reported comparable oncological outcomes [49] despite the higher costs and longer setup and procedure times associated with robotic systems [50].

Short-term outcomes from the ROLARR trial, which compared robotic and conventional laparoscopic approaches for rectal cancer, indicated no significant differences in conversion rates, margin positivity, mortality, complications, or postoperative dysfunction despite robotic procedures typically requiring more time and incurring higher costs [51]. A 2018 meta-analysis including ROLARR and other trials found similar perioperative outcomes and a lower likelihood of conversion to open surgery with robotic systems, though at the cost of longer operative times [52].

RALS enhances surgical precision through high-definition three-dimensional visualization and articulated instruments, which are particularly beneficial in challenging anatomical areas such as the narrow pelvis. The technology is designed to overcome some limitations of traditional laparoscopy, offering a stable camera platform and improved ergonomics for the surgeon [53]. Despite these advantages, systematic reviews and randomized trials have yet to demonstrate superior short-term clinical outcomes for robotic surgery over laparoscopy, though they suggest potential benefits for specific patient groups like those who are obese or undergoing sphincter-preserving procedures.

Limitations of RALS include its high cost, the steep learning curve for institutions and surgeons, and the lack of evidence showing a definitive advantage over laparoscopic surgery [54]. Long-term oncological outcomes between robotic and laparoscopic rectal surgeries are also still under evaluation, with studies like those by Cho et al. at Yonsei University showing no significant differences in recurrence rates or survival between methods [55]. Japanese studies have reported favorable long-term outcomes with robotic surgery for rectal cancer, although these results are from single-center, retrospective analyses and thus require cautious interpretation [56].

Overall, while robotic surgery has been established as safe and feasible for both benign and malignant colorectal conditions, its efficacy compared to traditional methods is still a subject of ongoing research. The increased prevalence of robotic procedures reflects a trend toward minimizing surgical invasiveness and optimizing patient outcomes, even though no clear benefits over laparoscopy have been definitively proven. The benefits of robotic surgery likely lie in its enhanced integration and interaction with artificial intelligence systems, augmented reality, and image-guided surgery [57,58]. Particularly, intraoperative ultrasound with specific robotic probes has recently shown promise in assisting with lesion characterization (malignant/benign) as well as surgical margin assessment. As the surgical community continues to evaluate RALS, particularly in complex colorectal applications, further studies will be crucial to define its role in future surgical practices [59].

## 4. Open vs. Laparoscopy vs. Robotic Approach

The TME technique was introduced in 1982 by Professor Heald, with well-known outstanding oncological results, at a time in scientific history when the open approach was the standard of care. Subsequently, as is now well known, mini-invasive techniques have established themselves in the surgical field as the preferred technique for numerous advantages in terms of quality of life and better and faster postoperative recovery but without clear data proving their oncological superiority.

In fact, this is confirmed by several studies that do not record a difference in terms of oncological radicality between the open, laparoscopic, and robotic techniques but highlight the improvements made by the minimally invasive surgery user in the patient’s postoperative course. Finally, Seow’s analysis shows that robotic TME achieved an improved distal resection margin distance and a shorter length of hospital stay compared to the other techniques. However, no other differences were observed in oncological or recovery parameters between open TME, laparoscopic TME, robotic TME, or TaTME [60].

### 4.1. Transanal Endoscopic Microsurgery (TEM)

TEM, pioneered by the German surgeon Gerhard Buess in 1983, has significantly advanced the surgical treatment of rectal diseases, particularly benign and early-stage malignant lesions [61]. This kind of MIS employs specialized microsurgical instruments, increasing precision in excision and providing better exposure, visualization, and access to higher rectal lesions than traditional methods [62].

Concerning TEM, the literature reports lower morbidity and quicker recovery compared to extensive transabdominal surgeries, although its adoption is conflicted by the costs of specialized equipment and a challenging learning curve [62]. However, it is highly effective for treating small, low-risk rectal carcinomas (G1/2, T1, and N0), offering advantages like reduced bleeding, less postoperative pain, and shorter hospitalizations [63].

The method achieves oncological outcomes similar to those of radical surgical procedures for selected early-stage lesions while significantly lowering both morbidity and mortality. TEM’s utility extends beyond tumor excision to the post-polypectomy management of carcinoid tumors, with studies reporting no recurrence [64]. Its applications also include resecting retrorectal cystic hamartomas, draining pelvic abscesses, and repairing rectourethral and rectovaginal fistulas [65].

TEM has become the preferred approach for removing large, sessile, or recurrent rectal adenomas that are unsuitable for endoscopic removal, showing better outcomes in terms of negative margins and lower recurrence rates compared to traditional TAE [66]. Its effectiveness in managing rectal malignancies was underscored in a meta-analysis comparing it favorably against TAE [67].

While neoadjuvant radiotherapy, followed by total TME, remains the gold standard for rectal cancer treatment, TEM offers a valuable alternative for cases where the quality of life concerns, such as incontinence or stoma necessity, are significant [68]. Guidelines currently advocate for the use of TEM in managing T1N0 rectal cancers, highlighting the benefits of the technique over traditional TAE in achieving more favorable resection margins and lower local recurrence rates [69,70]. A randomized study corroborated TEM’s equivalence to standard oncologic resection in local recurrence and survival rates, with superior postoperative outcomes [71].

However, treating T2N0 rectal cancers with TEM presents challenges, often showing higher local recurrence rates, especially in patients who are not candidates for TME. Furthermore, the Urbino trial explored the long-term oncological efficacy of TEM for T2N0 cancers, showing comparable disease-free survival rates to laparoscopic resection post-neoadjuvant chemoradiation, albeit with slightly higher local recurrence in the TEM group [70].

As clinical experience with TEM grows, its integration into rectal cancer treatment regimens expands from definitive therapy for early-stage cancers to a component of palliative care or multimodal treatment for advanced cases, reflecting its increasing recognition for efficacy and safety in treating a wide spectrum of rectal pathologies [71].

### 4.2. Transanal Minimally Invasive Surgery (TAMIS)

TAMIS was introduced in 2010 as a hybrid technique blending TEM and single-site laparoscopy. It offered a cost-effective alternative to the more expensive TEM, using a multichannel port and standard laparoscopic tools, demonstrating its viability in an initial series of six patients [72]. This technique uses a single-site port transanally along with conventional laparoscopic instruments, providing enhanced visualization and precision for the surgical resection of rectal lesions (Figure 2).

TAMIS offers several advantages over TEM, including shorter setup times, the use of readily available laparoscopic equipment, and a potentially shorter learning curve for surgeons already skilled in laparoscopic procedures. It achieves superior surgical outcomes with lower rates of specimen fragmentation and positive resection margins than standard transanal excision, with studies reporting only a 4% fragmentation rate and a 6% incidence of microscopic margin positivity [73].

This approach is applicable to a range of conditions, such as benign adenomas, neuroendocrine tumors under 2 cm, and well-differentiated T1 invasive carcinomas smaller than 3 cm. It is especially suited for early rectal cancers classified as a T1 stage, where it can serve both as a diagnostic biopsy and a curative procedure based on pathological results. For T1 cancers with favorable pathologic features, TAMIS might be the only treatment required, while those with higher-risk features might still need radical resection or adjuvant therapy [74].

For more advanced stages like T3 or in cases where patients have nodal disease or distant metastases, TAMIS is used to confirm the T stage and inform further treatment strategies, possibly including chemoradiation. This role highlights the importance of integrating TAMIS into a multidisciplinary treatment plan to optimize patient outcomes.

In addition, TAMIS is beneficial for managing indeterminate T1 versus T2 lesions without clear nodal disease, serving as a crucial step in definitive biopsy processes. If pathology indicates a T1 lesion with favorable characteristics, the procedure could be curative. Conversely, less favorable T1 or any T2 lesions may require more extensive surgical interventions [75].

However, in situations where patients are unfit for radical surgery due to medical conditions, TAMIS offers a palliative or exploratory option, minimizing the physiological impact of surgery while effectively managing local tumor control. This adaptability makes TAMIS particularly valuable for patients with significant comorbidities or those at high surgical risk [76].

Beyond oncological applications, TAMIS is employed for repairing rectourethral fistulas, removing rectal foreign bodies, and conducting complex resections in the lower gastrointestinal tract. Its versatility extends the scope of transanal surgeries, contributing significantly to modern surgical practices for rectal disease management. The continuous integration of TAMIS into clinical settings is supported by ongoing research and technological advancements, enhancing its safety, feasibility, and effectiveness for a wide array of rectal pathologies [77].

### 4.3. Transanal Total Mesorectal Excision (TaTME), Transanal Transection and Single-Stapled Anastomosis (TTSS), and Others

**Figure 6:**
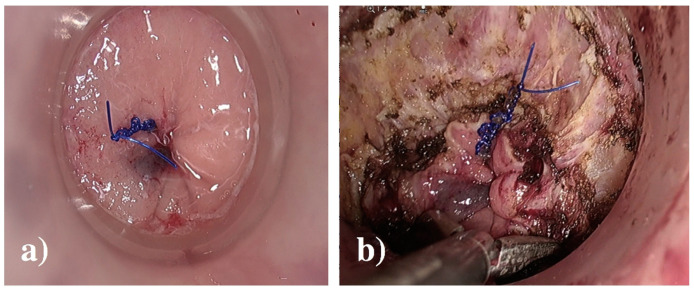
(**a**) Transanal prolene purstring with 1-0 prolene, (**b**) full-thickness rectotomy. From Larach JT et al. [78].

**Figure 7:**
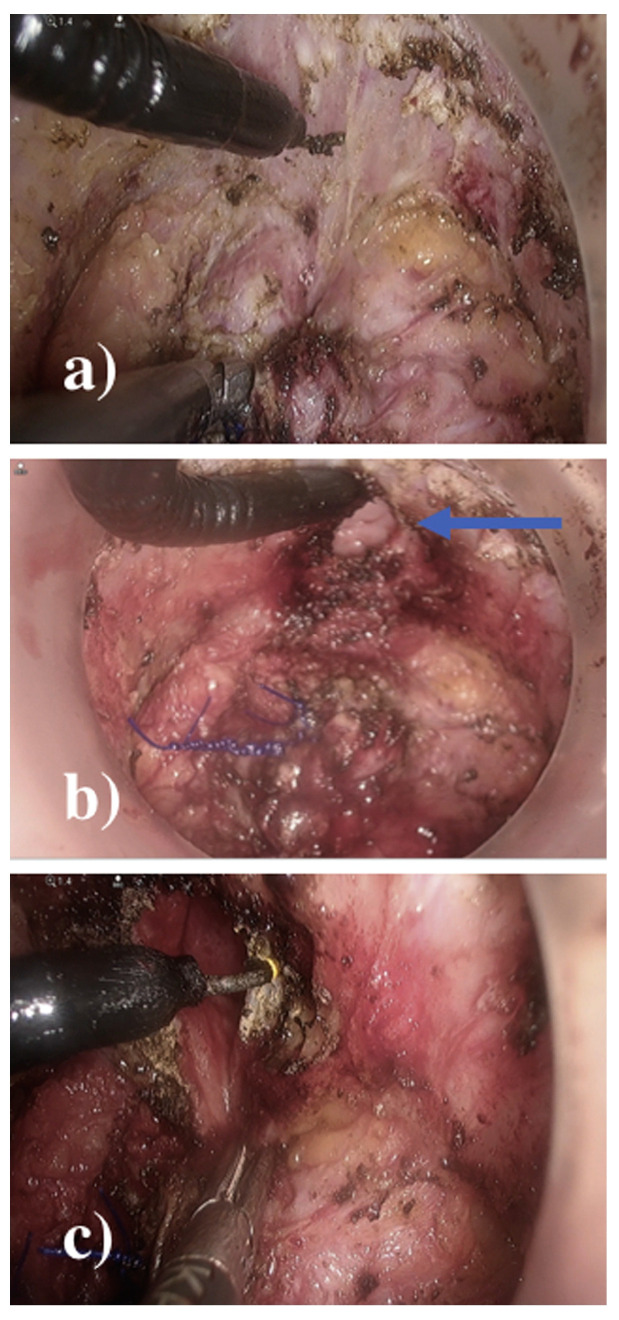
(**a**) Anterior dissection through rectovaginal septum, (**b**) posterior vaginal wall breakthrough at the inferior vaginal margin (blue arrow), and (**c**) dissection of vaginal stalks laterally and transanally, with hook diathermy. From Larach JT et al. [78].

TaTME (Figure 6 and Figure 7) [78], first described by Sylla et al. in 2010, represents a significant advancement in the surgical management of rectal cancer, particularly for challenging cases like lower rectal tumors, obese patients, or those with anatomical constraints, such as a narrow pelvis or prostatic hypertrophy [79]. This technique, which combines the principles of TME with transanal access, aims to enhance the precision of dissection and improve oncological outcomes by ensuring a clear circumferential resection margin (CRM), a crucial point in reducing local recurrence rates [30,80].

Indeed, TaTME is particularly advantageous because it provides a direct and magnified view of the surgical field, which is beneficial in difficult pelvic procedures. This method allows for better maneuverability and control of surgical instruments, potentially decreasing the rate of local recurrence and improving the QoL post-surgery by preserving urinary and sexual functions and reducing the need for permanent stomas [81,82]. The technique’s feasibility extends beyond oncological applications; it is also employed in benign diseases, such as proctectomy with ileal pouch–anal anastomosis, for conditions like UC or familial adenomatous polyposis, providing excellent cosmetic results [83]. Recent systematic reviews and cohort studies have stressed the promise of TaTME advantages, showing comparable morbidity and mortality rates to traditional laparoscopic TME, with the added benefits of reduced positive CRM and distal resection margin (DRM) rates. Moreover, high-quality TME specimens with clear margins have been consistently reported across studies, highlighting the procedure’s technical efficacy [83,84]. However, the approach does present challenges, such as the learning curve and the need for specialized training, especially in managing complex cases, like bulky tumors in the mid or low rectum, where TaTME can be especially beneficial.

Ongoing research and randomized controlled trials, such as the COLOR III trial, are expected to provide deeper insights into the long-term oncological outcomes of TaTME and further validate its role in modern rectal cancer surgery [84,85]. This growing body of evidence suggests that TaTME is a safe, effective, and innovative approach for both malignant and benign rectal diseases, expanding the surgical options available to colorectal surgeons and potentially setting new benchmarks in patient outcomes and QoL post-surgery [86].

**Figure 8:**
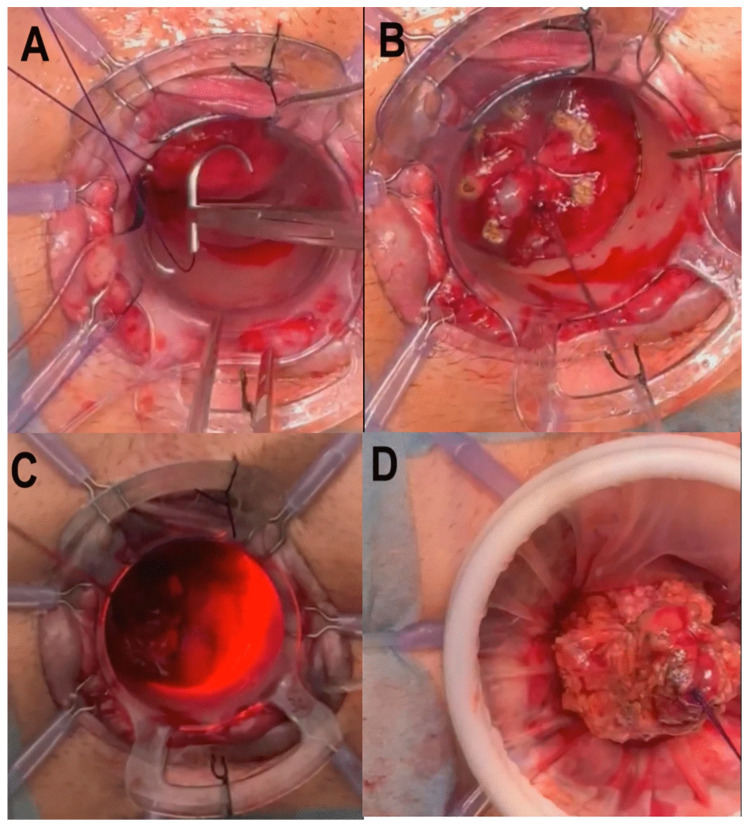
Steps of TTSS. (**A**) The first purse string (0/polypropylene) suture was placed distally to the tumor and transanally at a safe distance from the tumor. (**B**) The mucosa distal to the closure of the first tobacco pouch was marked. (**C**) Full-thickness circumferential rectotomy was performed using monopolar energy, facilitated by transillumination from the abdominal cavity. (**D**) Once the rectotomy was finished, a wound shield was placed before the sample was extracted, either transanally or transabdominally. From Vivas López A et al. [87].

**Figure 9:**
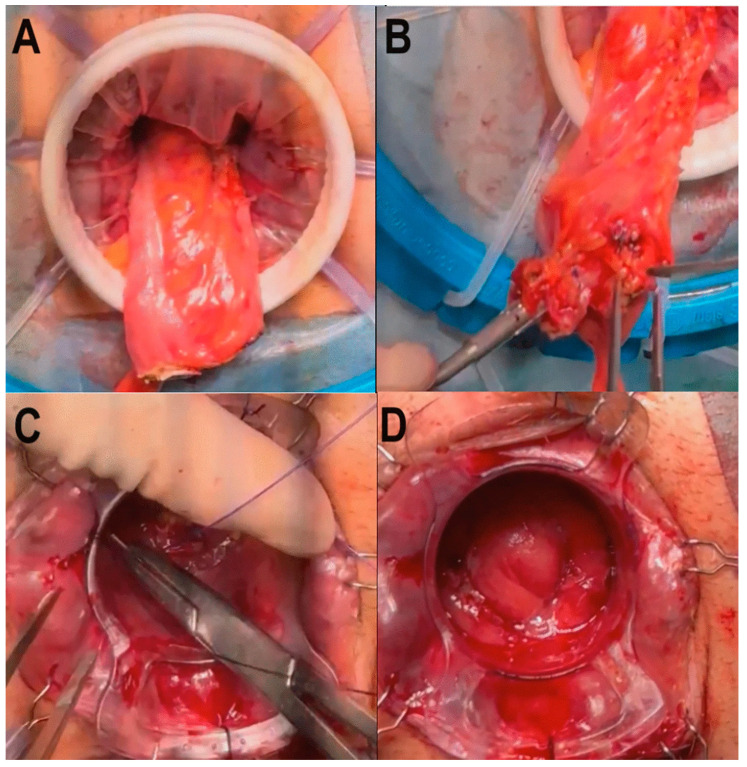
Steps of TTSS. (**A**) Completed section of the colon. (**B**) The circular anvil of the stapler (CSC-KOL^®^ 29 mm, B. Braun) was placed and secured in the proximal sectioned colon, and the colon was repositioned toward the pelvis. (**C**) The tractors of the Lone-Star^®^ were repositioned and placed at the level of the external margin. The 34 mm anoscope was repositioned again, and a second purse-string (0/polypropylene) suture was made, this time in the distal rectum. (**D**) The stapler was attached to the anvil, and a single-stapled anastomosis was created, with the anoscope still in place for optimal control. From Vivas López A et al. [87].

**Figure 10:**
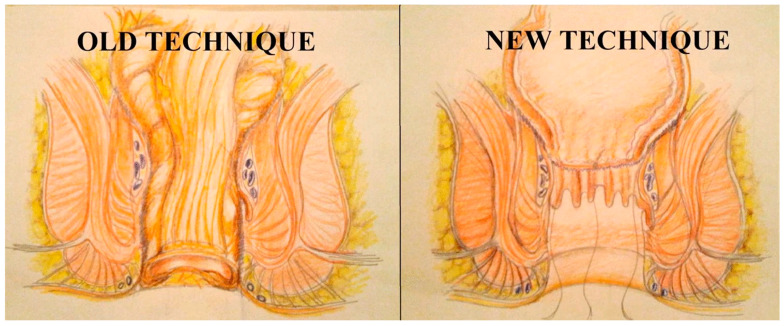
Schematic representation of the differences between the old and the new pull-through technique. From Bianco F et al. [89].

A new trend introduced by Spinelli et al. is the technique called transanal transection and single-stapled anastomosis (TTSS) (Figure 8 and Figure 9) [87], which is a surgical procedure that combines the laparoscopic abdominal approach with the perineal one. Indeed, in this technique, the rectum is transected transanally, allowing for direct visualization and enhanced control over margins of resection. After resection, the anastomosis is performed using a single circular stapler (single-stapled anastomosis). This technique ensures the precise approximation of tissue margins, reducing the risk of complications, such as anastomotic leaks. Moreover, TTSS has been shown to be particularly beneficial in cases where the tumor is located near the anal margin, where access is limited, and precision is crucial. Utilizing this technique can improve surgical outcomes and promote a better postoperative QoL for patients [88]. Recently, Bianco et al. [89] introduced and mastered the short stump and high anastomosis pull-through (SHiP) procedure for delayed coloanal anastomosis without a stoma in patients with low rectal cancer, modifying the concepts of the Turnbull–Cutait technique and reducing the complications rate, as well as improving patients’ QoL (Figure 10) [89]. A recent randomized multicenter controlled trial, comparing the two-stage Turnbull–Cutait pull-through hand-sewn coloanal anastomosis (*n* = 46) or standard hand-sewn coloanal anastomosis associated with diverting ileostomy (*n* = 46) in patients undergoing ultralow anterior rectal resection needing hand-sewn coloanal anastomosis, demonstrated that, after a 3-year follow-up, the modified delayed coloanal anastomosis is a valid alternative to the traditional in terms of morbidity, fecal continence, patient satisfaction, quality of life, and oncological outcomes, with a not-negligible benefit of avoiding a temporary stoma [90].

## 5. Watch and Wait

The “Watch and Wait” (W&W) strategy in rectal cancer aims to avoid unnecessary TME surgery, which carries significant risks, such as postoperative complications, functional impairment, and the potential need for a permanent stoma. Initially, this approach was thought to be suitable only for frail or elderly patients, but it is now considered for patients who achieve a complete clinical response (cCR) after neoadjuvant chemoradiotherapy (nCRT). W&W offers improved QoL by sparing patients the physical and functional consequences of surgery, though it carries a risk of local regrowth (25–38%), which can often be managed with salvage surgery. However, some studies report oncological outcomes and survival rates with W&W comparable to traditional surgery, especially when a sustained cCR is achieved. The key methods for identifying cCR include clinical examination, endoscopy, MRI, and PET/CT, with biopsies playing a less crucial role.

The W&W approach is particularly suited for patients with distal rectal tumors, where avoiding TME surgery can prevent stoma-related complications. Age is not a contraindication, and younger, fit patients can also benefit from W&W. Obviously, early detection of local regrowth is crucial for successful salvage surgery. Surveillance involves frequent digital rectal exams (DRE), endoscopies, and MRI scans every 8–12 weeks for the first 3 years to detect local regrowth, with less frequent follow-up after that [91].

## 6. Discussion

Rectal resection addresses a range of conditions, including cancer, IBD, endometriosis, and rectovaginal or rectourethral fistulas, often resulting in significant QoL challenges, such as loss of rectal reservoir function, urinary and sexual dysfunction, and pelvic pain. Despite advancements in surgical techniques from abdominoperineal resection (APR) with permanent colostomy to sphincter-saving procedures, patients frequently suffer from postoperative symptoms, like pelvic pain and defecatory dysfunctions, ranging from incontinence to constipation, reported in up to 90% of cases post-surgery. Moreover, sexual dysfunction post-treatment affects a substantial percentage of patients, with many reported ceasing of sexual activity [88].

The introduction of laparoscopic surgery offered reduced blood loss, pain, and shorter hospital stays, yet the long-term oncological outcomes and QoL benefits appear equivalent to open surgery. Initial improvements in QoL and lower rates of sexual and urinary dysfunctions with laparoscopic methods tend to diminish over time [92,93,94]. Studies, including the CLASICC trial, indicate similar long-term QoL outcomes between laparoscopic and open approaches [95].

In this context, robotic surgery has emerged as an alternative, showing potential for less postoperative pain and reduced sexual dysfunction, though higher costs and longer operation times pose ongoing challenges [25]. TaTME has gained traction for its precise dissection capabilities in lower rectal cancer, potentially lowering conversion and leakage rates and morbidity, although some studies suggest better fecal incontinence and overall QoL outcomes with laparoscopic approaches [96]. Technological advancement and innovation in surgery are making previously impossible treatments a reality, such as the management of metastatic CRC tumors. These developments have transformed the landscape of cancer treatment, allowing for more precise and effective interventions [97].

In IBD management, surgeries aim to minimize trauma due to the potential need for multiple interventions. Up to 80% of CD and 30% of UC patients may require surgery during their disease course. MIS, preferred for its reduced recovery times and complication rates, still presents challenges in cases of complicated or recurrent disease [98]. Emerging techniques, like robotic-assisted surgery and natural orifice specimen extraction, are becoming more prevalent, though conclusive data on their efficacy is still required [99].

TaTME, since its introduction, demonstrated promising outcomes in recent studies, suggesting potential advantages over traditional approaches in terms of CRM positivity and TME quality. However, comprehensive data to confirm these benefits and establish long-term oncological superiority are still needed [100]. Indeed, the largest single series included 140 patients who underwent TaTME, showing promising results, with no conversions and comparable operative complications, though the international registry suggests the need for cautious interpretation of these results due to the reported variability in outcomes and procedure integrity [101,102].

## 7. Future Directions

Single-port laparoscopic approaches present technical challenges due to limited workspace, frequent instrument collisions, and loss of triangulation, leading to restricted ergonomics and a steep learning curve that have hindered widespread adoption. Robotic systems with flexible and articulated instruments offer the potential to overcome these limitations, restoring dexterity and triangulation for both transparietal and natural orifice procedures. Single-port surgery demonstrates promising safety and effectiveness in colorectal cancer with high rates of successful oncological resection, adequate lymph node retrieval, and minimal intraoperative blood loss [103].

Recent advancements in robotic surgery have introduced multi-articulated and flexible devices capable of intraluminal and transluminal procedures with precision comparable to conventional surgery [104]. In rectal cancer, robotic transanal minimally invasive surgery (R-TAMIS) has demonstrated better complication rates, margin negativity, and recurrence outcomes compared to the published Lap-TAMIS series. Nonetheless, comparative studies with traditional laparoscopic approaches remain scarce, and only a few studies have employed flexible robotic platforms like the Medrobotics Flex System [105].

The emergence of new robotic systems, such as Endoquest Robotics and the Anovo Surgical System, designed specifically for natural orifice procedures, is expected to expand possibilities in this field. Additionally, the integration of advanced software into robotic platforms could accelerate the adoption of extended reality, computer vision, artificial intelligence, and image-guided surgery, offering significant improvements in both surgical training and patient outcomes [106,107].

## 8. Conclusions

Global trends show an increase in rectal cancer cases, necessitating advancements in diagnosis and treatment to maintain high-quality care and improve patient QoL. Techniques like TAMIS and TaTME are emerging as viable alternatives to the traditional open TME with neoadjuvant or adjuvant therapy. Robotic surgery is proving to be a safe alternative to laparoscopic approaches, offering the potential for further enhancements in surgical outcomes. MIS is increasingly adopted for managing IBD due to its superior outcomes, although the learning curve remains a challenge. Surgeons in training should be exposed to multidisciplinary care to ensure optimal patient management. Finally, the continuous evolution in rectal surgery highlights the importance of integrating transanal approaches and the need for ongoing advancements in surgical techniques to enhance patient care.
